# ADHD and attentional control: Impaired segregation of task positive and task negative brain networks

**DOI:** 10.1162/netn_a_00034

**Published:** 2018-06-01

**Authors:** Brian D. Mills, Oscar Miranda-Dominguez, Kathryn L. Mills, Eric Earl, Michaela Cordova, Julia Painter, Sarah L. Karalunas, Joel T. Nigg, Damien A. Fair

**Affiliations:** Department of Behavioral Neuroscience, Oregon Health and Science University, Portland, OR, USA; Department of Behavioral Neuroscience, Oregon Health and Science University, Portland, OR, USA; Department of Behavioral Neuroscience, Oregon Health and Science University, Portland, OR, USA; Department of Behavioral Neuroscience, Oregon Health and Science University, Portland, OR, USA; Department of Behavioral Neuroscience, Oregon Health and Science University, Portland, OR, USA; Department of Behavioral Neuroscience, Oregon Health and Science University, Portland, OR, USA; Department of Psychiatry, Oregon Health and Science University, Portland, OR, USA; Department of Behavioral Neuroscience, Oregon Health and Science University, Portland, OR, USA; Department of Psychiatry, Oregon Health and Science University, Portland, OR, USA; Department of Behavioral Neuroscience, Oregon Health and Science University, Portland, OR, USA; Department of Behavioral Neuroscience, Oregon Health and Science University, Portland, OR, USA; Advanced Imaging Research Center, Oregon Health and Science University, Portland, OR, USA; Department of Psychiatry, Oregon Health and Science University, Portland, OR, USA

**Keywords:** ADHD, Functional connectivity MRI, Attentional control, Default mode network, Task positive network, Negative connectivity

## Abstract

In children with attention deficit hyperactivity disorder (ADHD) difficulty maintaining task focus may relate to the coordinated, negatively correlated activity between brain networks that support the initiation and maintenance of task sets (task positive networks) and networks that mediate internally directed processes (i.e., the default mode network). Here, resting-state functional connectivity MRI between these networks was examined in ADHD, across development, and in relation to attention. Children with ADHD had reduced negative connectivity between task positive and task negative networks (*p* = 0.002). Connectivity continues to become more negative between these networks throughout development (7–15 years of age) in children with ADHD (*p* = 0.005). Regardless of group status, females had increased negative connectivity (*p* = 0.003). In regards to attentional performance, the ADHD group had poorer signal detection (d′) on the continuous performance task (CPT) (*p* < 0.0001), more so on easy than difficult d′ trials (*p* < 0.0001). The reduced negative connectivity in children with ADHD also relates to their attention, where increased negative connectivity is related to better performance on the d′ measure of the CPT (*p* = 0.008). These results highlight and further strengthen prior reports underscoring the role of segregated system integrity in ADHD.

## Author Summary

Maintaining task focus has been thought to relate to the coordinated activity between brain networks that support the initiation and maintenance of task sets (task positive networks) and networks that mediate internally directed processes (i.e., the default mode network). Here we find that segregation between these functional networks is impaired in children with ADHD, shows developmental lag, and is related to attentional impairments as measured by the continuous performance task. These results highlight and further strengthen prior reports underscoring the role of segregated system integrity in ADHD and its relationship to impairments in attention.

## INTRODUCTION

Attention deficit hyperactivity disorder (ADHD) is a major public health concern that affects an estimated 5% of children in the United States (Boyle et al., 2011; Faraone et al., 2015). Children with ADHD have impairments in attention that are manifested in real-world negative outcomes both in and out of the classroom (Biederman et al., 2004; Diamantopoulou, Rydell, Thorell, & Bohlin, 2007; Miller & Hinshaw, 2010). Several lines of evidence suggest that aspects of resting-state functional brain connectivity MRI (rs-fcMRI) are atypical in ADHD (Castellanos & Aoki, 2016; Costa Dias et al., 2013; Fair, Nigg, et al., 2012; Konrad & Eickhoff, 2010; Krain & Castellanos, 2006). In particular, prior theories have suggested that the absence of a negative correlation between task positive and task negative systems in the brain might relate to attentional issues in participants in ADHD (Castellanos et al., 2008; Hoekzema et al., 2014; Sonuga-Barke & Castellanos, 2007; Sripada, Kessler, & Angstadt, 2014; Sun et al., 2012). These brain systems are believed to be important for external and internal information processing, respectively (Dosenbach et al., 2007; Fox et al., 2005; J. D. Power, Fair, Schlaggar, & Petersen, 2010). The negative correlations between them have been shown to be reliable and robust across processing techniques (Fox, Zhang, Snyder, & Raichle, 2009; Hutchison et al., 2012; C. Keller et al., 2013; Kelly et al., 2008; Yan, Craddock, Zuo, Zang, & Milham, 2013), are related to modulations in local field potential power (Hutchison, Hashemi, Gati, Menon, & Everling, 2015; C. Keller et al., 2013; Popa, Popescu, & Pare, 2009), and may reflect the ongoing balance of specialization (and integration) between the distinct functional networks they are composed of (Fox et al., 2005; Hutchison et al., 2015).

In the current report, we characterize connectivity between internally oriented systems (i.e., the default mode network, DMN), and task positive, externally oriented networks, which include regions in the cingulo-opercular, salience, fronto-parietal, dorsal attention, and ventral attention networks (Dosenbach et al., 2007; Fox et al., 2005; J. D. Power et al., 2010). The DMN is a large and robustly replicable network that reduces its baseline level of activity during a task (Broyd et al., 2009; Buckner, Andrews-Hanna, & Schacter, 2008). In contrast, task positive networks increase their level of activity while engaging in tasks. These regions have been implicated in response selection, planning of willful acts, and preparing to respond to environmental events (Fransson, 2005, 2006). The task positive system is composed of several separate subnetworks including the fronto-parietal, cingulo-opercular, salience, dorsal attention, and ventral attention networks (Dosenbach et al., 2007; Fox et al., 2005; J. D. Power et al., 2010). There is a striking degree of negative correlation between the DMN and task positive networks that suggests a reciprocal relationship and a potential antagonism in terms of the psychological functions these networks serve (Cocchi, Zalesky, Fornito, & Mattingley, 2013; Fox et al., 2005; Fransson, 2005, 2006). In fact, when individuals transition from internally focused cognition to goal-directed, attentionally demanding behaviors, the DMN becomes more deactivated (Buckner et al., 2008).

The dynamics between these two networks not only may be important for characterizing ADHD, but may be a critical component of typical brain organization. Interestingly, in typically developing children negative correlations between the DMN and regions in the task positive network matures from childhood to early adulthood (7–21 years), and connections that show age-related effects also show larger ADHD group differences (Sripada, Kessler, & Angstadt, 2014). This suggests that ADHD may be characterized by dysmaturation between connections that continue to mature during development (Sripada, Kessler, & Angstadt, 2014). However, direct relationships between this system and age in ADHD has not been explored and it remains unclear whether the system changes in any group during early childhood and adolescence (7–15 years).

Although ADHD has been generally characterized by underconnectivity between a subset of connections between the DMN and task positive networks in ADHD (Castellanos et al., 2008; Castellanos & Aoki, 2016; Hoekzema et al., 2014; Sripada, Kessler, Fang, et al., 2014; Sun et al., 2012), findings are often connection specific, and the evidence remains inconclusive. Deficits in this system are thought to play a key role in ADHD-related dysfunction but are also potentially related to typical attentional processing. Kessler and colleagues showed that maturation of this system may be related to attentional performance in healthy controls (Kessler, Angstadt, & Sripada, 2016), but the role this system plays during earlier development and its direct relationship to failures in various aspects of attention in ADHD have not been tested.

Of course, attention is a multifaceted construct with the specific subcomponents varying somewhat by model (Fan & Posner, 2004; Posner & DiGirolamo, 1999). In the current report we focus on the relationship of negative connectivity between task positive and task negative networks and vigilance (which is related to the arousal function of attention). Vigilance is defined as the ability to maintain attentional focus to rare events (Riccio & Reynolds, 2006; Tucha et al., 2009). Vigilance is classically indexed by the continuous performance task (CPT), which requires an individual to respond to rare target events presented amid frequent nontarget events (Huang-Pollock, Karalunas, Tam, & Moore, 2012; Tucha et al., 2009). In many versions of the task used in ADHD populations, the difficulty of discriminating target and nontarget events is relatively easy (e.g., respond to the letter *X* but to no other letters), but it is also possible to manipulate the discrimination difficulty by including stimuli that are either more or less distinct from each other (Cornblatt, Risch, Faris, Friedman, & Erlenmeyer-Kimling, 1988; Huang-Pollock et al., 2012). There is also evidence that task difficulty may play a role with several studies finding larger ADHD performance differences on *easy* relative to *difficult* discriminations, interpreted as children with ADHD being less able to endogenously direct the focus of their attention and thus more reliant on task demands to exogenously engage attention (Friedman-Hill et al., 2010; Lenartowicz et al., 2014). Vigilance impairments are a prominent deficit in children with ADHD (Huang-Pollock et al., 2012; Tucha et al., 2009); however, the neural mechanisms of these attentional impairments in ADHD remain poorly understood.

This report studies a longitudinal sample of 432 subjects and 604 scans that were strictly controlled for movement during the MRI. We investigate connectivity patterns between task negative and task positive networks and their relationship with attentional processes utilizing an equivalent-pairs CPT, a widely used and validated measurement sensitive to task vigilance that also allows us to examine the relationship between brain networks and vigilance under varying discrimination difficulty (Cornblatt et al., 1988; Huang-Pollock et al., 2012). We test whether negative correlations between the DMN and task positive networks are reduced in children with ADHD, whether these negative correlations strengthen with development, and whether these connectivity patterns are related to attention measured by the CPT.

## METHODS AND MATERIALS

### Participants

This study included 502 participants with 816 MRI scans. Of these, a total of 432 children with 604 scans met all our motion censoring criteria (see below) and were included in the final analysis. The control group consisted of 176 children with a total of 231 total scans; 51 subjects had two scans and two subjects had three scans. The ADHD group had 256 subjects, 373 total scans; 85 subjects had two scans and 15 subjects had three scans. Children were between 7 and 11 years at baseline, and the lag design of approximately one year spans 7–15 years (average lag 1.51 years control, 1.62 years ADHD). The average age of the control group was 10.67 years (*SD* = 1.53) and included 89 males. The average age of the ADHD group was 11.05 (*SD* = 1.58) and consisted of 181 males. Sex and age were included as covariates and follow-up analyses matched the number of male and female scans included in each group. See the Supplementary Information (Mills et al., 2018) for recruitment and diagnostics.

### Human Connectome Project MRI Dataset

An independent dataset was used in order to define the connections that are most anticorrelated between the task positive and task negative networks. For this analysis we used data from the Human Connectome Project (HCP) consortium “500 Subject” release and included 61 healthy adult control subjects (22–35 years of age, 26 males), which were selected based on their optimal data quality and low motion (see Supplementary Information, Mills et al., 2018). These data were obtained from the publically available HCP database (https://db.humanconnectome.org).

### Behavioral Testing: Continuous Performance Task

At each scan date, a version of the identical pairs continuous performance task (IP-CPT) was used to examine vigilance and sustained attention (Cornblatt et al., 1988; Halperin, Sharma, Greenblatt, & Schwartz, 1991). An IP-CPT was used because it is less vulnerable to the ceiling effects that interfere with calculation of signal detection parameters in a less difficult CPT. The version used here was modeled on tasks used successfully in other studies of ADHD (Curko Kera, Marks, Berwid, Snatra, & Halperin, 2004). In the task, children viewed a series of four-digit numbers displayed one at a time in pseudorandom order to ensure unpredictability while achieving the required ratio of trial types. A total of 11 different four-digit numbers were used. When two identical numbers appeared back-to-back, the child pushed the response button. We used a 200 ms display followed by a 1,500 ms dark screen, for a total time per trial of 1,700 ms. Target frequency was 20%. Another 20% of trials were “catch” trials in which the back-to-back numbers differed by only one digit, creating a difficult discrimination, and 60% of trials were “stim” or “nontargets” in which subsequent numbers differed by multiple digits, making them comparatively easy discriminations. With a total of 300 stimuli, the task required about 10 min to complete. For both the easy (“stim”) and difficult (“catch”) trials, d′, an index of perceptual sensitivity (i.e., the ability to discriminate between target and noise; Stanislaw & Todorov, 1999), was calculated as our main index of vigilance (Cornblatt et al., 1988; Halperin et al., 1991). A higher d′ score indicates better performance and a greater sensitivity in distinguishing the target from the nontargets. D′ is typically interpreted as an index of vigilance (Sergeant, Oosterlaan, & van der Meere, 1999) and is commonly impaired in studies of ADHD (Huang-Pollock et al., 2012; Tucha et al., 2009; Willcutt, Doyle, Nigg, Faraone, & Pennington, 2005). See the Supplementary Information (Mills et al., 2018) for more detail.

### Functional Connectivity MRI Processing

Children were scanned on a Siemens Tim Trio 3.0 Tesla MRI scanner, consisting of 15 min of resting-state BOLD data acquired in three sequential 5-min runs. Strict motion censoring procedures were applied to resting-state functional maps, volumes with a framewise displacement (FD; Fair, Nigg, et al., 2012; J. Power, Barnes, Snyder, Schlaggar, & Petersen, 2012) exceeding 0.2 mm were excluded, and only subjects with greater than 4 min of remaining motion-free data were included in this analysis. For further motion quality control we removed an additional 30 scans whose average default to task positive connectivity was related to mean FD, thus ensuring that data quality passed criteria as recently described by Siegel et al. (2016, see Supplementary Information, Mills et al., 2018). Results are also presented without this secondary motion control (see Supplementary Information, Mills et al., 2018). In order to match the amount of data included on all scans, connectivity matrices were calculated with 4 min of randomly selected clean data for all scans. No differences in remaining FD were found between groups (*p* = 0.48). For data analysis, we selected 333 regions of interest (ROIs) with predefined network affiliation based on prior work (Gordon et al., 2014). These ROIs are in good agreement with known functional brain systems and have been assigned functional identities and network definitions. For all participants, the resting blood oxygen level–dependent time series for each region of interest was cross-correlated (Pearson correlations) between the time series of all ROI pairs, yielding a correlation value that was then Fisher *r* to *z* transformed. The final result was a 333 ×333 size correlation matrix for each subject. MRI scan parameters, motion censoring, motion quality control, and preprocessing are described in full detail in the Supplementary Information (Mills et al., 2018).

### Network Definitions and Statistical Methods

**Figure 1.**
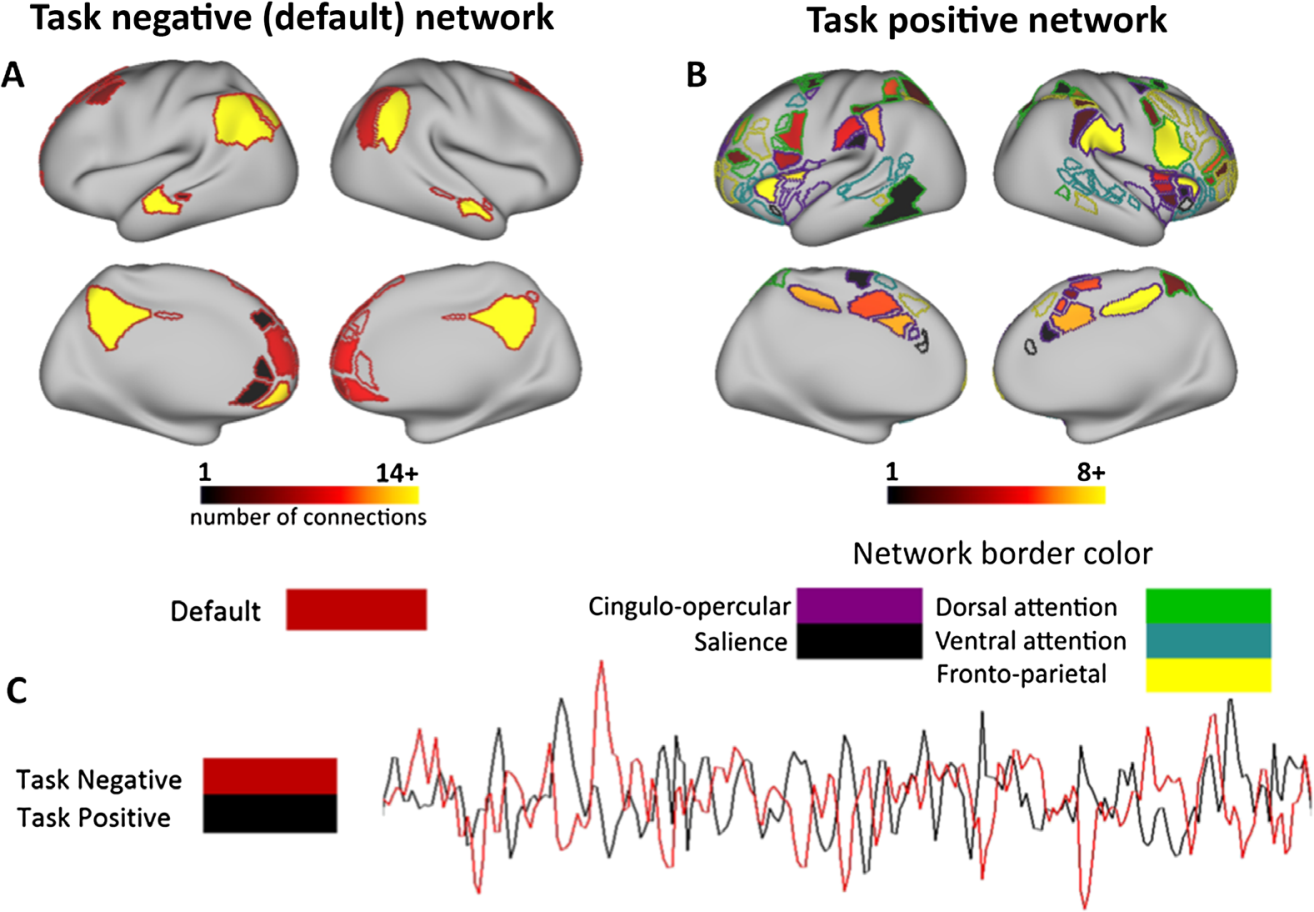
Regions with significant negative correlations between the (A) task negative and (B) task positive networks. For the main analyses, 250 negative connections between systems were analyzed (i.e., *r* < −0.35, 5% density). Regions are colored based on the importance of a given region to the network mask, where brighter colors represent regions with more negative connections between task positive and task negative systems. Unfilled regions do not have a connection within this network mask threshold. Borders of the regions are colored based on network assignment defined by Gordon et al. (2014). For illustrative purposes, (C) represents BOLD time series data from a randomly selected control subject depicting the negatively correlated relationship between a region of the task positive and task negative network.

Not all connections between task positive and task negative networks confer negative correlations (Cocchi et al., 2013). Therefore, an independent dataset of 61 adults was used to define the most highly negative connections between task negative (i.e., DMN) and task positive networks (i.e., fronto-parietal, cingulo-opercular, salience, dorsal attention, and ventral attention networks; Gordon et al., 2014). The average connectivity between networks was then computed and connections that were most highly anticorrelated were selected. For the main analyses connections with an *R* < −0.35 were used (250 negative connections of 5,043 total connections between default and task positive networks, corresponding to a connection density of 5%). See Figure 1 for these regions, their importance to the network mask, and their network assignments. Less stringent thresholds and network masks corresponding to the DMN to all negative connections, unrestricted to the task positive network, were also analyzed.

For the main analyses, connectivity between each of the 250 task positive and task negative connections was averaged for each scan. In each case, connectivity was then assessed for main effects of group status, sex, age, and their interactions, controlling for subject head motion (mean FD). If interaction terms are insignificant, only main effects will be modeled for subsequent analyses, unless otherwise stated. Statistics were computed using a linear mixed-effects model (R version 3.2.1; lme4 package). Individual parameter effects were estimated with the R package, lmerTest. Linear mixed effects modeling allows for an estimation of the fixed effects while incorporating the longitudinal nature of the data by including within-subject variation as nested random effects.

The R package MuMIn was used to evaluate the best fitting model and Akaike information criterion (AIC) was evaluated for model selection and to guard against overfitting. Statistical effects were evaluated with three models; Model 1 considered only main effects, Model 2 included a group by age interaction, and Model 3 was a full omnibus model including a three-way interaction between age, group, and sex (see Supplementary Table 3 for formulas; Mills et al., 2018). Model 1 had the lowest AIC and was used to test main effects and connection-level effects; Model 3 was used to evaluate whether there were any interaction effects; and Model 2 further tested significant interactions from Model 3, including the interaction between age and group status on connectivity.

Connection-level analyses separately modeled each of the 250 connections between networks and identified connections that were significantly (*p* < 0.05, false discovery rate [FDR] corrected; Benjamini & Hochberg, 1995) related to a parameter of interest (using Model 1). Results from these analyses are shown in Supplementary Table 4 (Mills et al., 2018). Given that even trend-level results likely influence the conglomerate measures of connectivity, brain images shown in Figures 3 and 4 were constructed as follows. Significant *t* scores (*p* < 0.05, uncorrected) for each connection were summed for each parameter effect of interest in a given direction (i.e., ADHD < control). This summed *t* score for each region then visualized on the cortical surface. This analysis depicts a region’s influence on each effect of interest.

In order to ensure our results were not driven by an imbalance in gender distribution between groups (ADHD = 241 male scans, control = 123 male scans, 1.96:1 ADHD:control ratio), we performed confirmatory analyses that matched the gender distribution between groups. For these analyses 123 ADHD male scans and 100 ADHD female scans were randomly selected (118 ADHD male scans and 8 ADHD female scans were randomly removed) in order to match the control distribution. Statistical models were refit with this matched subset and parameter effects were recomputed. This process was repeated 10,000 times, each time randomly selecting a subset of ADHD subjects, and the distribution of *p* values were averaged to obtain a gender-balanced effect for each effect of interest (age, group, gender, age by group interaction, and CPT by behavior).

## RESULTS

### Negative Correlations

An independent dataset of 61 adults was used to define the connections between task negative (default) and task positive networks that were most highly anticorrelated. Connections that survived a threshold of *R* < −0.35 (corresponding to the 5% strongest negative connections) were included in the network mask used for the main analyses. To ensure these effects were not threshold dependent, supplementary analyses tested anticorrelated network masks of *R* < −0.3 and *R* < −0.375 (see Supplementary Table 2, Mills et al., 2018). For the main analyses, we found 250 negative connections from the default mode; the majority were from the default to the cingulo-opercular networks (170 connections), followed by the dorsal attention network (76 connections), and a small number of connections with the fronto-parietal network (four connections, from two regions). No negative connections were found at this threshold between the default and ventral attention or salience networks. See Supplementary Table 1 (Mills et al., 2018) for the number of regions included in each network and the number of connections between each network. Follow-up analyses show a similar distribution of negative connections throughout the task positive network when default connections were unrestricted to task positive regions (see Supplementary Table 1, Mills et al., 2018).

A major aim of this study was to examine whether negative connectivity between task positive and task negative networks is distinct in children with ADHD, between sexes, and whether these connectivity patterns continue to mature across development. See Supplementary Table 3 (Mills et al., 2018) for the linear mixed-effects models to test these effects and their interactions. Model 1 (main effects only) was the best fitting model (*df* = 7, AIC = −1,011) followed by Model 2, which included all two-way interactions, (*df* = 8, AIC = −1,003), followed by Model 3 which also included a three-way interaction between group, age, and sex (*df* = 11, AIC = −979).

### Negative connectivity is greater in controls than in children with ADHD

**Figure 2.**
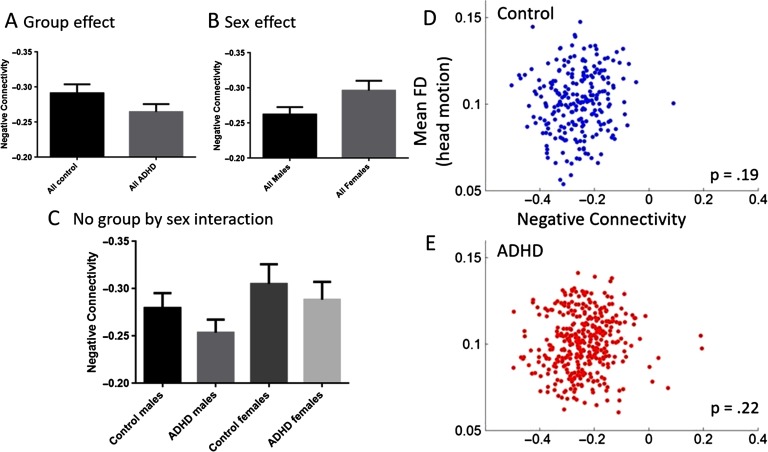
Relationships between average negative connectivity, group status, gender, and motion. (A) ADHD subjects have weaker negative connectivity between the task negative and task positive networks. (B) Across groups, females have increased negative connectivity. (C) The ADHD group difference is similar in males and females (i.e, no group by sex interaction). Given the longitudinal nature of the data, main effects and interactions were tested in a linear mixed-effects model. (D–E). Head motion, measured by mean remaining frame displacement (FD), is not related to average negative connectivity in either (D) control subjects (*r* = 0.08, *p* = 0.19) or (E) ADHD (*r* = 0.06, *p* = 0.22).

We found that ADHD subjects had reduced negative connectivity than control subjects. This was consistent across all three statistical models (Model 1; main effect of group status, *t* = 3.03, *p* = 0.002, TD *M* = −0.291, *SD* = 0.098, ADHD *M* = −0.264, *SD* = 0.109; see Figure 2, and Supplementary Table 3, Mills et al., 2018, for each statistical model). The main effect of group status was also robust to connection density when defining the most negative connections between task negative and positive networks (*R* < −0.3, − 0.35, − 0.375; see Supplementary Table 2, Mills et al., 2018), and the main effect of ADHD was preserved in analyses focusing on these alternative network masks, including those from the default network to the rest of the brain (see Supplementary Table 2, Mills et al., 2018). An additional analysis added CPT (d′ easy) as a covariate in the full model (Model 3). Although less significant than without including this measure in the model, group (*p* = 0.040) and sex (*p* = 0.044) effects remained significant. This suggests that these differences in connectivity are not purely due to vigilance deficits, but are also related to other aspects of ADHD and sex.

**Figure 3.**
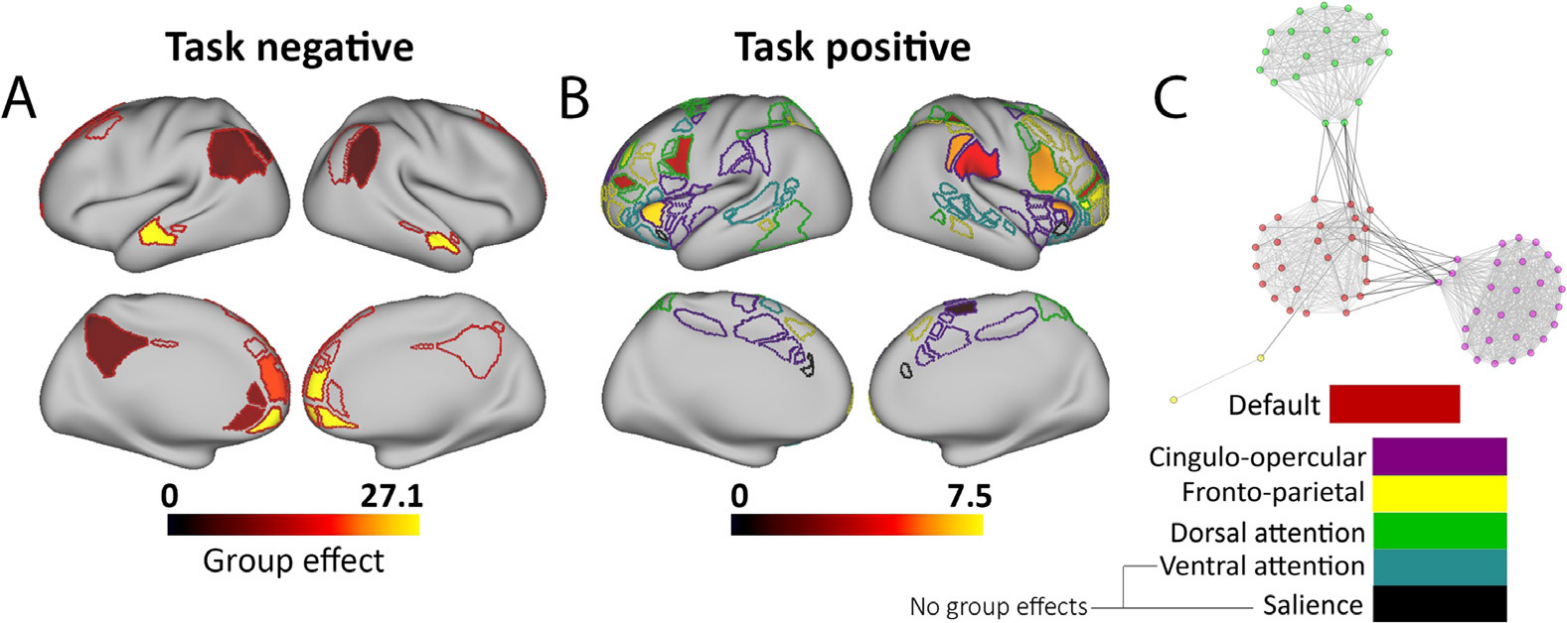
ADHD effects visualized on the brain. ADHD subjects have reduced negative connectivity between many connections of the task negative and task positive networks. The group effect (summed *t* scores for all group effects in a given region) are shown, where brighter colors indicate a region where connections are weaker in ADHD subjects than controls. Connections are visualized between the (A) task negative and (B) task positive network. (C) A statistical distance metric visualizes the number and origin of differences in negative connectivity between groups. Nodes that are closer to the center are connected by solid black lines and denote a significant group effect from a given task positive network to the task negative network (default mode in the center). Note that many of the group effects that show weaker negative connectivity are between the default network and the cingulo-opercular and dorsal attention network.

In order to examine which connections were likely to drive effects in the main analyses, the individual connections with the group effects (*p* < 0.05, uncorrected) are visualized in Figure 3, and each connection that shows a group effect that passes an FDR-corrected *p* < 0.05 is listed in Supplementary Table 4 (Mills et al., 2018).

### Negative connectivity is greater in females than in males

Across groups, girls had greater negative connectivity than boys (*t* = −2.93, *p* = 0.003; see Figure 2B). When examining only control subjects, this sex effect nearly reached statistical significance (*p* = 0.056). We found no group by sex interaction (*p* > 0.5; see Figure 2C). This suggests that the ADHD effect is similar for males and females and that the increased negative connectivity observed in females is independent of diagnosis.

### Negative connectivity is related to age in children with ADHD

**Figure 4.**
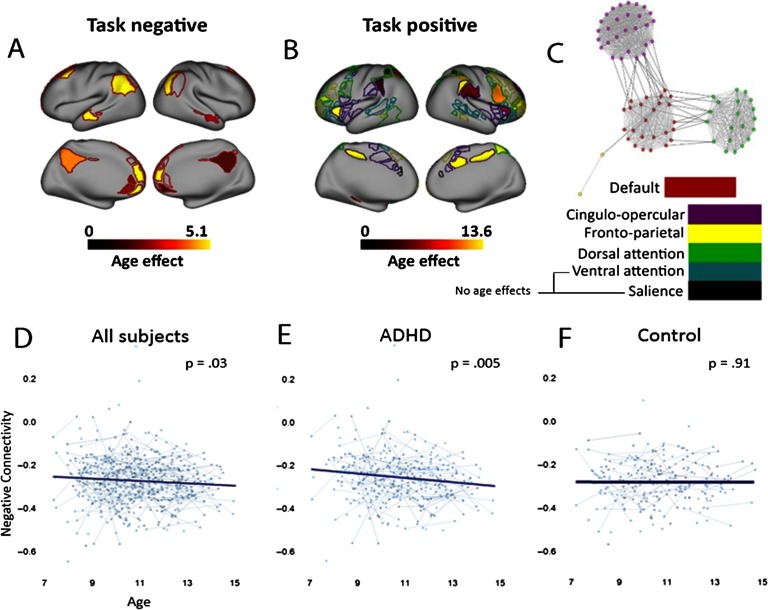
Connectivity between the task positive and task negative network becomes increasingly negative with age. The relative age effects (summed *t* scores for all age effects in a given region) are shown for regions within the (A) task negative and (B) task positive network. Brighter colors indicate regions with more age effects, where negative connectivity increases with age. (C) Solid black lines denote a significant age effect from a given region within the task positive network, to a region within the task negative network. (D) Averaged negative connectivity between networks decreases with age, across both groups. This effect is driven by a significant relationship between age and connectivity in (E) ADHD rather than (F) control subjects.

In order to assess whether this system continues to mature through development, a main effect of age shows that negative connectivity continues to increase with age (main effect of age Model 1, *t* = −2.249, *p* = 0.025). However, we also found a trend towards an age by group interaction (Model 3, *t* = −1.91, *p* = 0.057; Model 2, *t* = −1.836, *p* = 0.06), where children with ADHD showed a stronger age by connectivity relationship (age parameter effect with only ADHD subjects, *t* = −2.76, *p* = 0.005) than controls (only control subjects, *t* = 1.01, *p* = 0.91). This suggests that the effect of age may be driven by children with ADHD (see Figures 4D, 4E, and 4F). No other interaction term reached significance.

Overall, these findings suggest that negative connectivity between task positive and task negative networks is decreased in children with ADHD, that negative connectivity is greater in females regardless of group status, and that negative connectivity continues to increase in children with ADHD.

### Effect of Head Motion on Connectivity and Behavior

**Figure 5.**
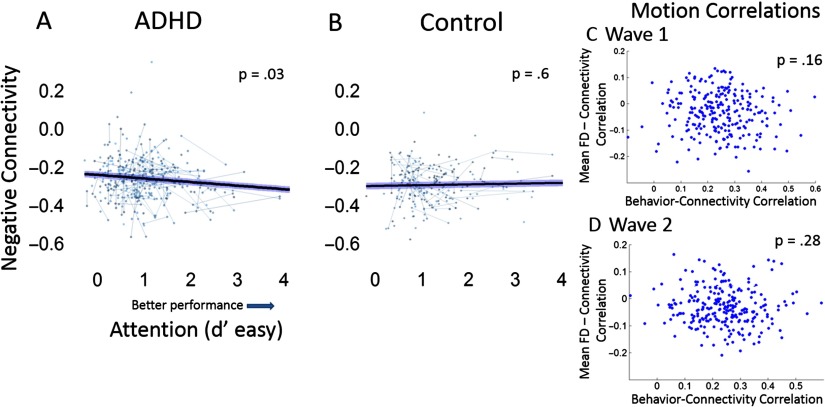
Attention as measured by the CPT (d′ easy low-discrimination trials) is related to negative connectivity between task positive and negative networks in subjects with ADHD. (A) In ADHD subjects, greater negative connectivity between networks is related to better attentional performance on the CPT (*t* = −2.12, *p* = 0.03). (B) No relationship is found in TD children (*t* = 0.61, *p* = 0.60). Each dot represents a scan and gray lines connect longitudinal scans for each subject. 95% confidence intervals around the slope are shown. Importantly, the behavior connectivity relationship is not related to in scanner head motion at either behavioral and MRI scan (C) Wave 1 (*p* = 0.16) or (D) Wave 2 (*p* = 0.28).

Importantly, head motion, a major confound in neuroimaging studies, was not related to the average connectivity metric or average connectivity by behavior findings (see Figures 2D and 2E and Figures 5C and 5D). Age was also unrelated to mean remaining FD (*R* = −0.042, *p* = 0.3). Results are also robust to connection density of the network mask (see Supplementary Information for *r* < −0.3 and *r* < −0.375; Mills et al., 2018) and are maintained without the inclusion of mean FD as a model covariate (data not shown); these relationships are also maintained after the reinclusion of the 30 scans filtered based on the secondary motion-censoring criteria (see Supplementary Figure 1, Mills et al., 2018).

### Gender-Balanced Analyses

Finally, in order to address the gender imbalance in our sample (male subjects ∼2:1 ADHD: control), a permutation approach was taken to randomly select subjects from the ADHD group to match the gender distribution in the control sample. *P* values for each parameter effect were then averaged across 10,000 permutations to assess each effect’s significance. When modeling the main effects (Model 1), group status (*p* = 0.0081) and gender (*p* = 0.0083) remained significantly related to negative connectivity. After balancing the distribution of genders between groups, the main effect of age is no longer evident across groups (*p* = 0.187), but may be driven by a trend-level effect in ADHD subjects (group by age interaction effect, Model 2, *p* = 0.087).

### Connection-Level Analyses

Each of the analyses above focused on main effects on an averaged metric of negative connectivity between task positive and task negative networks. In order to examine the particular connections driving this effect, we next assessed the individual connections driving group, sex, and age effects. A list of connections between these networks (250-connection network mask), which showed significant main effects of group, age, and sex, are listed in Supplementary Table 4 (Mills et al., 2018). Significance was calculated based on a *p* < 0.05 (FDR corrected) using Model 1. Of note is that an abundance of connections influencing the group and age effects are found between the default mode and dorsal attention and cingulo-opercular networks, and that only connections between default and the cingulo-opercular drive gender effects.

### Behavioral Results

As expected, we found that children with ADHD had worse performance on the d′ measure of the CPT, an index assessing attentional vigilance (group effect controlling for age, sex, and task difficulty, *p* < 0.0001). We also found that performance on the CPT improved with age (*p* < 0.0001). Importantly, we found a group by task difficulty interaction (interaction effect controlling for age and sex (*p* < 0.0001), where ADHD children had worse performance than controls on easy discrimination trials (TD *M* = 2.76, *SD* = 0.84; ADHD *M* = 2.21, *SD* = 0.93) than on difficult trails (TD *M* = 1.34, *SD* = 0.79; ADHD *M* = 1.01, *SD* = 0.69). Although children with ADHD were impaired on both easy and difficult discriminations, the larger difference on easy trials suggests that children with ADHD have more attentional difficulties when task demands are low. No other interactions were significant, and gender was unrelated to performance on the CPT (*p* = 0.61).

### Brain Behavior Relationships

In a repeated measures analyses including all subjects and both d′ measures of task difficulty (i.e., easy and difficult trials), more negative connectivity was related to better attentional performance (*p* = 0.0064). This relationship was similar for easy and difficult trials (interaction effect between connectivity and d′ difficulty, *p* = 0.162), suggesting that connectivity was related to both difficult (*B* = −1.33, *t* = −2.11, *p* = 0.038) and easy (*B* = −2.81, *t* = −3.362, *p* = 0.008) d′ measures (all analyses controlled for mean FD). This relationship was not moderated by age or sex. However, a trend was observed where ADHD subjects showed a stronger relationship between average connectivity and the d′ (easy) than controls (group by behavior interaction *p* = 0.072). Post hoc analyses in the control group show no relationship between connectivity and d′ easy (*p* = 0.603) but do show a significant relationship when only analyzing ADHD subjects (*p* = 0.033). Difficult d′ conditions are trend level in the ADHD group (*p* = 0.103) and nonsignificant in control subjects (*p* = 0.342). Taken together this suggests that the relationship between easy discriminability trials on the CPT and average connectivity is driven by subjects with ADHD. These findings were also unrelated to motion (see Figures 5C and 5D), and the CPT-connectivity relationship is conserved with the inclusion of the 30 scans filtered based on the secondary motion-censoring criteria (see Supplementary Information, Mills et al., 2018). In the gender-balanced analyses, we found a significant relationship between CPT and negative connectivity in the easy (*p* = 0.045) but not difficult trials (*p* = 0.13). In the gender-balanced analysis, the CPT-connectivity relationship was only somewhat moderated by group status (*p* = 0.11), potentially because of a decrease in statistical power.

## DISCUSSION

Negatively correlated activity between task positive and task negative brain networks has previously been proposed to support attentional processes (Castellanos & Aoki, 2016; Weissman, Warner, & Woldorff, 2004). This study extends an emerging body of literature suggesting that negative functional connectivity between this system is a locus of dysfunction in ADHD and shows developmental lag in ADHD. It also demonstrates that these networks are negatively connected in females regardless of group status and that dysfunction of this system may be related to attentional impairments in childhood ADHD.

### There Are Several Benefits of the Current Study Relative to the State of the Literature

Reduced negative connectivity between negative and task positive networks in ADHD was a robust finding across statistical models, with and without matching gender distributions in our sample, and was significant across a range of connection densities. Although other studies have found reduced negative connectivity between particular task positive to task negative connections (Castellanos et al., 2008; Castellanos & Aoki, 2016; Hoekzema et al., 2014; Sripada, Kessler, Fang, et al., 2014; Sun et al., 2012), this study benefited from an independent and well-curated dataset that defined the set of the most highly negative connections between these well-defined networks. This network mask was then used, at various thresholds, to compute an average connectivity metric for each scan. This allowed for a direct and straightforward assessment of the effect of age, sex, their interactions, and relationships to behavior. This was all done without suffering from issues dealing with multiple comparisons and easing interpretability of these effects. That being said, the work also highlights particular connections and networks that may be most perturbed within this averaged connectivity metric. These include a number of connections showing reduced negative connectivity in ADHD between the default, cingulo-opercular, and dorsal attention networks. It should be noted, however, that the particular connection and network effects may be biased towards the number of ROIs within each network, and these effects may shift based on connection density used in anticorrelated network mask and may change based on parcellation scheme. Nonetheless, the general trends found here are unlikely to be affected by such factors.

Another strength of this study is the large longitudinal sample collected at a single site. To the best of our knowledge, previous literature examining similar group effects have generally consisted of around 20 subjects per group (Castellanos et al., 2008; Hoekzema et al., 2014; Sun et al., 2012), with the exception of one large multisite study (Sripada, Kessler, & Angstadt, 2014). Although multisite studies can be powerful in their own right, many potential confounds can arise when combining subjects across studies and across sites, including differences in MRI acquisition protocols and data quality (Abraham et al., 2017; Friedman, Hastie, & Tibshirani, 2008), differences in participant instructions (e.g., eyes open vs. eyes closed; Yan et al., 2013), as well as differences in recruitment strategy and methods for diagnoses (Abraham et al., 2017). Even if differences between sites are known, they can be difficult to accurately model, and can make it more challenging to isolate the true relationships of the variable of interest. Our study avoids these issues because it is large and homogenous in all of its procedures, and ridged diagnoses. This does not mean, of course, that other multisite studies are not useful or helpful, or that they don’t have their own advantages, but having a large homogenous sample for scientific purposes is a strong positive of the current study. Another strength of this study is in its use of the human connectome processing pipeline, which has many advantages including more accurate spatial localization and improved signal to noise (Smith et al., 2013; Van Essen et al., 2012). The current study also uses the most up-to-date and stringent motion correction criteria, an important confound particularly in developmental studies (J. Power et al., 2012; Yerys et al., 2009). These safeguards helped ensure that both group differences as well as imaging-behavior relationships (Siegel et al., 2016) were unrelated to in-scanner head motion.

### Negative Connectivity Continues to Develop in Children With ADHD

As stated previously, children with ADHD showed a significant relationship between average negative connectivity and age, and no such relationship was found in typical children during this age bracket (7–15 years). These findings might suggest a few things. The first is that in typical children, this system is developed by early childhood and will not continue to mature into the early teen years. While there are several pieces of literature that would suggest the continued maturation of this system (J. Keller et al., 2015; Kelly et al., 2008; Luna, Marek, Larsen, Tervo-Clemmens, & Chahal, 2015; Marek et al., 2015; Sato et al., 2016; Spreng, Stevens, Viviano, & Schacter, 2016; Sripada, Kessler, & Angstadt, 2014; Sun et al., 2012), strict experimental controls on this large of a functional dataset in ADHD is the first of its kind, and thus might run counter to prior reports. Moreover, we are referring here to findings related to average connectivity between these systems, and individual connections may show developmental maturation between these ages in typical controls. It remains possible that individual connections increase in negative connectivity throughout typical development, but that these are subtler changes, or more variable across connections. This would result in differences that would be averaged out in our single metric of negative connectivity and appear as a lack of development of this system in typical controls.

Under this context, in regards to the significant age relationship in children with ADHD, our findings might suggest the underdevelopment of this system relative to controls, and thus, their negative connectivity continues to mature during 7–15 years of age. Consistent with some prior reports (El-Sayed, Larsson, Persson, Santosh, & Rydelius, 2003; Shaw et al., 2007; Sripada, Kessler, & Angstadt, 2014), this would hint at a maturational lag. The findings and this interpretation are also consistent with others who have found that individual negative connections that show ADHD effects are also the connections that show continued development with age (Sripada, Kessler, & Angstadt, 2014). As larger datasets are acquired and children are tracked, future research should directly assess developmental lag in ADHD across the life span.

### Females Have Increased Negative Connectivity Compared With Males

Interestingly, across groups, females had increased negative connectivity between the DMN and task positive networks. Boys are over twice as likely to be diagnosed with ADHD as girls (Boyle et al., 2011), and this led to an imbalance in the proportion of males in the ADHD group compared with the control group (∼2:1). Importantly, our results were robust to permutation tests randomly matching groups by gender distribution. It has been reported that females have greater connectivity within the DMN (Allen et al., 2011; Filippi et al., 2013; Tomasi & Volkow, 2012); however, studies of attention networks are mixed (Allen et al., 2011; Bluhm et al., 2008; Filippi et al., 2013; Scheinost et al., 2015). To the best of our knowledge, this work is the first to show that negative connectivity from the DMN to task positive system may also be an important locus of sex-dependent differences in brain connectivity during early childhood. Although females with ADHD had greater negative connectivity compared with males with ADHD, the relative decrease compared with controls was the same for both genders. That being said, ADHD females had a similar degree of negative connectivity as control males. It is challenging to speculate whether the similar blunting of this network signifies a shared mechanism for ADHD across genders, or whether the relatively intact network structure in ADHD females suggests a distinct etiology and presentation of ADHD. That being said, decreased baseline connectivity between these networks in males could represent a vulnerability for males to develop attentional difficulties, and potentially contribute to the 2:1 male bias in ADHD. These preliminary speculations highlight an avenue for future research to explore.

### Attentional Performance Is Decreased in ADHD and Is Related to Negative Connectivity

As expected, we found that children with ADHD show impairments on attentional vigilance as measured by the CPT. These deficits were greater in easy discrimination trials, compared with more difficult discrimination trials. This is in line with previous research showing that attention performance may improve in ADHD with increasing cognitive load (Forster & Lavie, 2016; Friedman-Hill et al., 2010; Lenartowicz et al., 2014) and suggests that children with ADHD may show deficits in attentional filtering particularly when task demands are low, although evidence is mixed (Friedman-Hill et al., 2010; Huang-Pollock et al., 2005).

Importantly, reduced negative connectivity was related to worse vigilance, specifically in children with ADHD. This suggests that impaired development of this system may contribute to the observed problems in attention. No relationships were found in typical children, which could be due to a number of reasons. One possible reason is that it is unlikely that this system is the only driver of performance on the CPT; rather, when this system is highly atypical it can disrupt performance on this task. It is also possible that given a more difficult task, correlations between these resting-state networks and attention may emerge, even within the typical variation seen in controls. Importantly, not all connections between the default mode and task positive networks confer negative correlations. Here we select only the most consistently negative connections defined in an independent dataset. The selection of these connections may be somewhat restrictive and could potentially shift based on transitory brain states that are not captured during a rest (Cocchi et al., 2013). Therefore, these relationships could potentially change if sampled throughout all connections between these networks rather than only from the most consistently anticorrelated connections between task positive and negative networks. Last, we note that ADHD is a vastly heterogeneous disorder encompassing different cognitive (Fair, Bathula, Nikolas, & Nigg, 2012; Karalunas et al., 2014) as well as neural phenotypes (Costa Dias et al., 2013; Gates, Molenaar, Iyer, Nigg, & Fair, 2014). Just as importantly, the control population is also heterogeneous in a similar fashion (Fair, Bathula et al., 2012). It is possible that performance on the CPT and its relationship to negative connectivity might vary relative to various cognitive profiles, which might be skewed within the control and ADHD samples. Further work will be needed to clarify such possibilities, and to refine whether this network is implicated in deficits in a diverse set of attentional mechanisms, such as alerting, orienting, or top-down attention (Posner & Petersen, 1990), or whether it selectively supports only one domain of attention.

## CONCLUSIONS

In summary, in a large cohort of children with ADHD with strict motion correction criteria, negative connectivity between task positive and task negative networks is reduced in ADHD, continues to develop with age, and is related to impairments in attentional vigilance. These findings highlight a potential neurophenotype for at least a subset of children with ADHD and serves as a potential marker of attentional impairments in this population.

## AUTHOR CONTRIBUTIONS

Brian D. Mills: Conceptualization; Data curation; Formal analysis; Investigation; Methodology; Project administration; Validation; Visualization; Writing – original draft; Writing – review & editing. Oscar Miranda-Dominguez: Data curation; Methodology; Software. Kathryn Mills: Methodology; Writing – review & editing. Eric Earl: Data curation; Writing – review & editing. Michaela Cordova: Data curation; Investigation. Julia Painter: Data curation; Investigation. Sarah L. Karalunas: Conceptualization; Methodology; Writing – review & editing. Joel T. Nigg: Funding acquisition; Investigation; Methodology; Project administration; Resources; Writing – review & editing. Damien A. Fair: Conceptualization; Funding acquisition; Investigation; Methodology; Project administration; Resources; Software; Supervision; Writing – review & editing.

## FUNDING INFORMATION

Funding for this project comes from the grants MH086654 (Nigg, Fair), MH096773 (Fair), MH099064 (Nigg), and MH091238 (Fair). Data were also provided (in part) by the Human Connectome Project, WU-Minn Consortium (principal investigators: David Van Essen and Kamil Ugurbil; 1U54MH091657) funded by the 16 NIH Institutes and Centers that support the NIH Blueprint for Neuroscience Research; OHSU Fellowship for Diversity and Inclusion in Research Program (Oscar Miranda-Dominguez); and by the McDonnell Center for Systems Neuroscience at Washington University.

## TECHNICAL TERMS

Resting-state functional connectivity: An fMRI method for mapping correlated patterns in spontaneous blood oxygen level dependent signal across the brain during wakeful rest.
Default mode network: Also referred to as the task negative network. The default mode is a collection of brain regions whose activities are highly correlated during rest; during goal-directed tasks the network reduces its baseline level of activity.
Task positive network: A collection of brain networks that during goal-directed tasks typically increase in baseline level of activity.
Continuous performance task (CPT): A behavioral measure of attentional vigilance, which requires an individual to respond to rare target events presented amid frequent nontarget events.
