# Supplementary material for: ADHD and attentional control: Impaired segregation of task positive and task negative brain networks
Source: Netw Neurosci. 2018 Jun 1;2(2):200–17. doi: 10.1162/netn_a_00034 (PMC6130439; doi:10.1162/netn_a_00034)
Supplement: Supplementary file 1 [file netn-02-200-s001.pdf]

Mills, B. D., Miranda-Dominguez, O., Mills, K., Earl, E., Cordova, M., Painter, J., Karalunas, S. L., Nigg, J. T., & Fair, D. A. (2017). Supplemental Materials for "ADHD and attentional control: Impaired segregation of task positive and task negative brain networks." *Network Neuroscience, 2*(2), 200-217.
https://doi.org/10.1162/netn_a_00034

Supplementary Materials:
Recruitment and Diagnostics

Children were recruited from families who volunteered in response to mass mailings in the community. Their diagnostic grouping was carefully evaluated in best-estimate, multi-stage case finding procedure that included parent clinical interview using the Kiddie Schedule for Affective Disorders and Schizophrenia (K-SADS-E) (Orvaschel, Lewinsohn, & Seeley, 1995) and parent and teacher standardized rating scales including the Conners' Rating Scale, 3rd edition (Conners, 2008), ADHD Rating Scale (DuPaul, Power, Anastopoulos, & Reid, 1998), and Strengths and Difficulties Questionnaire (Goodman, 1997). Intelligence was estimated with a three-subtest short form (Block Design, Vocabulary, and Information) of the Wechsler Intelligence Scale for Children, 4th edition (Wechsler, 2003), and academic achievement with word reading and numerical operations subtests of the Wechsler Individual Achievement test. A best-estimate diagnostic team reviewed this information, as well as IQ, academic scores, and observer notes, and independently assigned a diagnosis. Their agreement on ADHD/non-ADHD status was acceptable ($k > 0.85$ for all diagnoses occurring at base rate > 5% in the sample, including ADHD and ADHD subtype). Disagreements that could not be resolved by discussion would lead to exclusion, but in this study consensus could be achieved in each case, thus no subject had to be excluded for this reason.

Children were excluded if they did not meet criteria for ADHD or non-ADHD groups; if they had evidence of tic disorder, psychotic disorder, bipolar disorder, autism spectrum disorder, or mental retardation; if parent reported history of neurological illness, chronic medical problems, sensorimotor handicap, or significant head trauma (with loss of consciousness); or if they were taking psychotropic medications other than psychostimulants. Children were also excluded if they presented metal in their bodies, which could contra-indicate MRI acquisition or cause imaging artifacts (e.g. dental braces, intracranial aneurysm clips). Additional exclusion criteria for control children were: presence of conduct disorder, current major depressive episode, or ADHD. Only right-handed children were included in the present study. Children prescribed psychostimulant medications were scanned after a minimum washout period of five half-lives (i.e. 24–48 h depending on the preparation).

The Human Investigation Review Board at Oregon Health & Science University approved the research. Written informed consent was obtained from respective parents and written assent was obtained from all child participants.

Behavioral testing
Continuous performance task

An IP-CPT was used because it is less vulnerable to the ceiling effects that interfere with calculation of signal detection parameters in a less difficult CPT. The version used here was modeled on tasks used successfully in other studies of ADHD (Curko Kera, Marks, Berwid, Snatra, & Halperin, 2004). In the task, children viewed a series of four-digit numbers displayed one at a time in pseudo-random order to ensure unpredictability while achieving the required ratio of trial types. A total of 11 different 4-digit were used. When two identical numbers appear back-to-back, the child pushed the response button. We used a 200 ms display followed by a 1500 ms dark screen, for a total time per trial of 1700 ms. Target frequency was 20%. Another 20% of trials were "catch" trials in which the back-to-back numbers differed by only one digit, creating a difficult discrimination and 60% of trials were "stim" or "non-targets" in which

subsequent numbers differed by multiple digits, making them comparatively easy discriminations. With a total of 300 stimuli, the task required about 10 minutes to complete. For both the easy ("stim") and difficult ("catch") trials, d', an index of perceptual sensitivity(Stanislaw & Todorov, 1999) (i.e., the ability to discriminate between target and noise) and was calculated as our main index of vigilance (Cornblatt, Risch, Faris, Friedman, & Erlenmeyer-Kimling, 1988; Halperin, Sharma, Greenblatt, & Schwartz, 1991). A higher d' score indicates better performance and a greater sensitivity in distinguishing the target from the non-targets. D' is typically interpreted as an index of vigilance(Sergeant, Oosterlaan, & van der Meere, 1999) and is commonly impaired in studies of ADHD(Huang-Pollock, Karalunas, Tam, & Moore, 2012; Tucha et al., 2009; Willcutt, Doyle, Nigg, Faraone, & Pennington, 2005).

Mathematically, d' = ABS(HR) – ABS(FAR) and lnβ = LN[ORD(HR)/ORD(FAR)]. Where ABS(p) and ORD(p) = the abscissa and ordinates of the hit rate (HR) and false alarm rate (FAR) on a standardized normal distribution. For cases in which HR = 1 or FAR = 0, abscissa and ordinates are undefined so that d' and β cannot be calculated. Therefore, following Davies & Parasuraman (Parasuraman & Davies, 1977), when HR = 1 or FAR = 0, HR was recalculated as $2^{-1/\#\ \text{targets}}$ and FAR recalculated as $1\text{-}2^{-1/\#\text{non-targets}}$ in that event.

To ensure data quality we required the following filters. First, each block was considered valid if children had no more than 50% false alarms on the "easy" non-target trials in that block. Next average scores were computed for hit rate, omissions, false alarms on "easy" non-target trials, and false alarms on "difficult" or "catch" trials using all of the valid blocks. To be included in final analyses, children were required to have an average of >10% correct hits *and* <90% false alarms on "easy" non-target trials.

MRI data acquisition:

OHSU cohort: Participants were scanned on a Siemens Tim Trio 3.0 Tesla MRI scanner at the Advanced Imaging Research Center at OHSU. Structural images were obtained using a T1-weighted MP-RAGE sequence (TR=2.3s, TE=3.58ms, flip angle = 10°, TI = 900ms, voxel size = 1mm^3, 160 sagittal slices). A T2-weighted sequences was also acquired (TR = 3.2s, TE = 497ms, voxel size = 1mm, slices = 160) as well as magnitude and phase field maps to correct for geometric distortions due to susceptibility artifact. Resting-state functional BOLD images were acquired using a gradient-echo, echo-planar sequence (TR = 2500 ms, TE = 30 ms; FOV = 240 mm; flip angle = 90°; 3.75x3.75x3.8 mm). Full brain coverage was obtained with 36 contiguous interleaved 3.8 mm axial slices acquired parallel to the plane transecting the anterior and posterior commissure.

Human connectome project (HCP) cohort: Participants for the HCP related analyses were obtained from the HCP consortium "500 Subject release". These data are publically available on the human connectome project database (https://db.humanconnectome.org). Of these subjects we included 61 healthy control subjects (22-35 years of age, 26 males) which were selected based on their optimal data quality and low motion (at least 800 frames remained after motion scrubbing). All subjects included were unrelated. HCP data was acquired on a 3T Siemens Skyra optimized to achieve 100 mT/m gradient strength. All the data was corrected to account for the non-linearities associated with the high gradient and the displacement of the isocenter in this optimized system. For further details see the HCP 500 Subjects + MEG2 Data Release: Reference Manual (WU-Minn, 2014) and (Glasser et al., 2013). Two separate T1-weighted images were acquired and averaged, with a TR=2400 ms, TE=2.14 ms, TI = 1000 ms, FA = 8°,

and ES = 7.6 ms. Two T2-weighted images were acquired and averaged with a TR=3200 ms, TE=565 ms. T1-weighted and T2-weighted images were acquired with a voxel resolution of 0.7 mm (isotropic). Resting state BOLD data were acquired using a gradient echo echo planar imaging sequences with 2mm3 voxels, TR=720ms, TE = 33.1ms, and a multiband acceleration factor of 8. The HCP dataset included two sequential days of scanning in which two resting state scans were acquired on each day. Each of these EPI scans were acquired in both the left to right and right to left acquisition direction. In order to maximize the amount of data, all four datasets, both session (day 1 and day 2) and each acquisition direction, were processed as described above and timecourses were concatenated before construction of correlation matrices.

<u>MRI data processing</u>

OHSU sample**:** Data were processed using the pipelines from the Human Connectome Project (Glasser et al., 2013), which include the use of FSL (Jenkinson, Beckmann, Behrens, Woolrich, & Smith, 2012; Smith et al., 2004) and FreeSurfer tools (Desikan et al., 2006; Fischl & Dale, 2000; Fischl, Sereno, & Dale, 1999). Briefly, gradient distortion corrected T1-weighted and T2-weighted volumes were first aligned to the MNI's AC-PC axis and then non-linearly normalized to the MNI atlas. Later, the T1w and T2w volumes are re-registered using boundary based registration (Greve & Fischl, 2009) to improve alignment. Then, the brain is segmented using recon-all from FreeSurfer. Segmentations are improved by using the enhanced white matter-pial surface contrast of the T2-weighted sequence. The BOLD data is corrected for field distortions (using FSL's TOPUP) and processed by doing a preliminary 6 degrees of freedom linear registration to the first frame. After this initial alignment, the average frame is calculated and used as final reference. Next, the BOLD data is registered to this final reference and to the T1-weighted volume, all in one single step, by concatenating all the individual registrations into a single registration. Strict motion correction procedures were applied to resting state functional maps and volumes with a framewise displacement (FD) (Fair et al., 2012; Power, Barnes, Snyder, Schlaggar, & Petersen, 2012) which exceeded .2mm were excluded and only subjects with greater than 4 minutes of remaining motion free data were included in this analysis. In order to insure that the same amount of data was used in all subjects, 73 motion free frames were randomly selected to construct each scans covariance matrix.

<u>Surface registration</u>. The cortical ribbon defined by the structural T1-weighted and T2-weighted volumes is used to define a high resolution mesh which will be used for surface registration of the BOLD data. This cortical ribbon is also used to quantify the partial contribution of each voxel in the BOLD data in surface registration. Timecourses in the cortical mesh are calculated by obtaining the weighted average of the voxels neighboring each vertex within the grid, where the weights are given by the average number of voxels wholly or partially within the cortical ribbon. Voxels with high coefficient of variation, indicating difficulty with tissue assignment or containing large blood vessels, are excluded. Next, the resulting timecourses in this mesh are downsampled into a standard space of anchor points (grayordinates), which were defined in the brain atlas and mapped uniquely to each participant's brain after smoothing them with a 2mm full-width-half-max Gaussian filter. Subcortical regions are treated and registered as volumes. Two thirds of the grayordinates are vertices located in the cortical ribbon while the remaining grayordinates are subcortical voxels.

Nuisance regression. Additional preprocessing consists of regressing out the grey matter, ventricle and white matter average signal, and the movement between frames from the six image alignment parameters x, y, z, θ_x , θ_y, and θ_z on the actual and the previous TR and their squares, which correspond to the Volterra series expansion of motion (Friston, Williams, Howard, Frackowiak, & Turner, 1996; Power et al., 2014, 2012). The regression's coefficients (beta weights) are calculated solely based on frames with low movement, but regression is calculated considering all the frames to preserve temporal order in the data for filtering in the time domain. Next, timecourses are filtered using a first order Butterworth band pass filter to preserve frequencies between 9 and 80 mHz.

HCP sample: For HCP subjects, BOLD data was denoised using ICA-FIX a tool which uses independent Component Analysis (ICA) to account for nuisance and covariates. ICA-FIX automatically removes artifactual or "bad" components. Briefly, each voxel's timecourses from 25 HCP subjects were decomposed into 229 spatial components. Of these, on average 24 components were hand-classified as "good" and the remainder as "bad". Next, a classifier was trained to identify "good" and "bad" components. Once the classifier was optimized (by leave-one-subject-out cross validation), the resulting classifier was used to identify the "bad" components from each participant. Such components were removed by regressing the "bad" components (timecourses) out from the timecourses on each grayordinate. In addition to ICA-FIX BOLD data was further denoised by regressing the whole brain signal. All other processing techniques were identical between the HCP and OHSU cohorts.

Motion and whole brain regression

This study is the first to examine negative connectivity patterns in ADHD after recent realizations by the field of the critical importance of motion correction (Grayson et al., 2016; Power et al., 2013, 2012; T. Satterthwaite et al., 2012). In order to ensure that our findings were not driven by differences in head motion we use multiple preprocessing techniques aimed at eliminating motion effects, including motion regression, motion censoring based on frame displacement, and whole brain regression. Here, we use a motion cut off of FD < .2mm in order to maximize the amount of quality data, while excluding motion related artifacts in our sample. While motion is always a concern, particularly in hyperkinetic and developmental samples, the likelihood of our results being related to motion artifacts low given that the final sample was matched on average frame displacement.

As an additional quality control measure whole brain regression (WBR) was used. Outside of unique data collection circumstances, WBR has repeatedly been shown to reduce noise unlikely related to neural activity, remove cardiac and respiratory signals known to correlate with the global signal and reduce motion artifact (Grayson et al., 2016; K Murphy & Fox, 2016; Power et al., 2012; T. D. Satterthwaite et al., 2013; Schölvinck, Maier, Ye, Duyn, & Leopold, 2010). With this said, we recognize that there is still controversy regarding the use of WBR. While prior work has highlighted the benefits of this procedure (Grayson et al., 2016; Keller et al., 2013; Miranda-Domínguez et al., 2014; Power et al., 2014), there are others who disagree (Gotts et al., 2013). On the one hand it is clear that WBR reduces noise (Grayson et al., 2016; Power et al., 2012; T. D. Satterthwaite et al., 2013) and is one of the only methods which are able to correct for non-spatially dependent artifacts. Further, after WBR the structure of the negatively correlated networks are maintained in their spatial distribution and cross subject consistency (Fox, Zhang, Snyder, & Raichle, 2009). On the other hand, WBR can artificially

induce some low probability negative correlations (Kevin Murphy, Birn, Handwerker, Jones, & Bandettini, 2009). However, in our prior work we have confirmed that the rank order of the strongest negative and positive correlations does not overtly change with and without the use of WBR (Miranda-Domínguez et al., 2014). In addition, the strongest negative correlations have been validated with non-MR measures of brain activity (Keller et al., 2013). The current study examines only the strongest negative correlations which are of the highest probability of being the true and biological relevant negative correlations which are not artificially induced by the use of WBR. In total, our preprocessing and analyses ensure that the cleanest motion free data was used to calculate connectivity on only the most robust negative connections between networks.

Secondary motion censoring methods

It has recently become clear that links between connectivity and behavior may be influenced by subject head motion even after stringent motion correction and censoring methods (Siegel et al., 2016). Therefore, we ensured that 1) connectivity patterns were not related to subject head motion, and 2) that brain behavior correlations were not related to motion. In brief, the first step involved ensuring the correlation between average negative connectivity was not related to subjects head motion measured by mean remaining FD. Next, we calculated the motion influence on the functional connectivity-behavior relationship as described in full elsewhere (Siegel et al., 2016). In brief, this required that we ensure there was no correlation between the mean FD-connectivity relationship and the observed brain-behavior relationships. This was calculated as a "correlation of correlations", that is the correlation between the correlation of mean FD and all connections within the network mask, and the CPT-connectivity correlation. In short, we found modest relationships between head motion and these two parameters (see supplementary results) and so in order to ensure that our results were not influenced by subject motion our results were tested in multiple ways, step 1) With and without the removal of 30 scans whose behavior and or connectivity patterns were related to head motion (mean FD) and 2) with and without mean FD as a covariate in the linear mixed effects models.

Supplementary Results:

Main effects without secondary motion correction

Our main analyses removed 30 scans whose connectivity patterns were driving a modest relationship between head motion and functional connectivity, as well as been head motion and subject age. After their removal our analyses passed motion criteria defined by Siegel and colleagues (Siegel et al., 2016), see figure S1 for these scans. Without the exclusion of these subjects our effects were conserved. ADHD subjects had less negative connectivity ($p = .005$), and the age relationship ($p = .015$), and sex effect ($p = .021$) remained significant. The group by age effect remained a trend ($p = .09$). CPT relationships were also significant with these subjects included for the d-prime difficult ($t=-2.86$, $p = .004$), CPT easy condition ($t = -3.21$, $p = .001$) and the relationship remained stronger in ADHD subjects than controls with a significant interaction between d-prime difficult and group status ($p = .035$).

Connection level effects

In order to examine whether age, group, and sex effects were seen at the level of individual connections between the task positive and negative networks a similar set of analyses computed a linear mixed effects model for each connection between networks. Of the 250 connections between networks, 24 connections were significantly under-connected in ADHD

(FDR corrected $p < .05$), 15 connections showed significant age effects (greater negative connectivity and increased age), and 20 connections were more negatively connected in females. No effects (FDR corrected) were in the opposite direction (i.e. ADHD greater negative connectivity than controls). Each of these connections can be seen in Supplementary Table 4.

Supplementary Figures

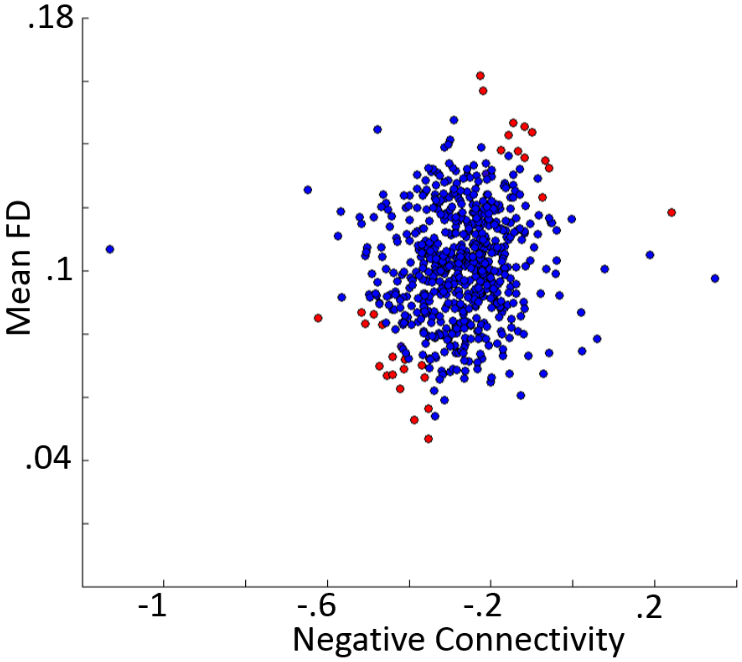

Supplementary Figure 1. Scans removed from the main analyses do to a relationship between motion and negative connectivity. Average negative connectivity between networks is shown on the x axis and scan remaining frame displacement (FD) is shown on the y axis. 30 scans were removed (red) which contributed to the motion, connectivity relationship. With the inclusion of the scans in red the FD-connectivity correlation ($r = .13$, $p < .0001$), without the scans included ($r = .06$, $p = .10$). Main effects (ie, group, age, sex effects, and CPT relationships) are conserved with the inclusion of these scans. The scan with a negative connectivity value below -1 (fischers transformed correlations) was not removed in the main analyses because it did not influence this connectivity-motion relationship, however, the exclusion of this data point (this scan was an ADHD subject) does not alter the significance of any results.

Supplementary Tables

Network	Number of ROIs in each network	Adult Network Mask (R<-.35) Main analyses	Adult Mask all connections (R < -.35)
Default	41	250	256
DorsalAttn	32	76	76
VentralAttn	23	0	0
CinguloOperc	40	170	170
FrontoParietal	24	4	4
Salience	4	0	0
Visual	39	NA	5
Supp Motor - mouth	8	NA	1

Supplementary Table 1. Number of ROIs per task positive – task negative network mask. Column 1. The number of ROIs in each network, as defined by Gordon et al.(Gordon et al., 2016). Column 2. Number of significantly negative connections (average R < -.35) between task negative (default) and task positive network regions, defined in an independent set of adults. Note the majority of negative connections are from the default to the cinguloopercular network. Column 3. Number of significantly negative connections between the default network and all other brain regions. Note that only two additional regions which are outside the task positive network (visual and supplementary motor) are now included in this network mask.

Method:	Network Mask R threshold	Number of Default Regions Included	Group difference (p-value)	Average Network Connectivity TD	Average Network Connectivity ADHD	Connectivity-Mean FD Correlation ADHD group (p-value)	Connectivity-Mean FD Correlation TD group (p-value)
Main subject list (with 30 scan removal) - Main Analysis	-0.35	250	0.0026	-0.29	-0.26	0.22	0.20
Addition of scans removed due to motion correlation (30 scans)	-0.35	250	0.0068	-0.29	-0.27	0.01	0.02
Main subject list (with 30 scan removal)	-0.375	155	0.0009	-0.30	-0.27	0.32	0.20
Main subject list (with 30 scan removal)	-0.3	547	0.0051	-0.26	-0.24	0.09	0.18
Analyses to all regions (not restricted to task positive regions)							
Main subject list	-0.35	256	0.0024	-0.29	-0.26	0.20	0.18
Main subject list	-0.375	157	0.0009	-0.30	-0.27	0.30	0.18
Main subject list	-0.3	591	0.0054	-0.26	-0.24	0.06	0.18

Supplementary Table 2. Alternative thresholds for defining the network mask. An independent sample of adults were used to define the connections which were most negatively correlated with the default mode. Main analyses considered connections from the default mode which were below R < -.35, and excluded 30 scans whose connectivity was correlated with motion (mean FD). Alternative analyses are shown at various thresholds, with and without the removal of 30 scans.

1) $Y = \beta_0 + \beta_1 \, x \, FD + \beta_2 \, x \, age + \beta_3 \, x \, group + \beta_4 \, x \, gender + \varepsilon$

$$2)\ Y = \beta_0 + \beta_1\, x\, FD + \beta_2\, x\, age + \beta_3\, x\, group + \beta_4\, x\, gender + \beta_5\, x\, (group\ x\ age) + \varepsilon$$

$$3)\ Y = \beta_0 + \beta_1\, x\, FD + \beta_2\, x\, age + \beta_3\, x\, group + \beta_4\, x\, gender + \beta_5\, x\, (group\ x\ age) + \beta_6\, x\, (group\ x\ age) + \beta_7\, x\, (gender\ x\ age) + \beta_8\, x\, (group\ x\ gender) + \beta_9\, x\, (group\ x\ age\ x\ gender) + \varepsilon$$

Supplementary Table 3. Statistical Models predicting average negative connectivity (Y). Model 1. Used for the main analyses to test main effects of age, group, and gender. Model 2. Used to test the age by group interaction. Model 3. Omnibus model testing the influence of all interactions between average connectivity and demographic variables. All models included mean remaining FD at each scan.

Group Effects		Age Effects		Sex Effects	
Connection	p-value	Connection	p-value	Connection	p-value
116_L_Default -- 155_L_DorsalAttn	0.00015	157_L_Default -- 189_R_DorsalAttn	0.00267	162_R_Default -- 223_R_CinguloOperc	0.03437
278_R_Default -- 155_L_DorsalAttn	0.00082	150_L_Default -- 236_R_DorsalAttn	0.00285	94_L_Default -- 245_R_CinguloOperc	0.04798
279_R_Default -- 249_R_CinguloOperc	0.00104	127_L_Default -- 168_R_FrontoParietal	0.00398	290_R_Default -- 187_R_CinguloOperc	0.04150
127_L_Default -- 155_L_DorsalAttn	0.00095	322_R_Default -- 110_L_DorsalAttn	0.00437	315_R_Default -- 84_L_CinguloOperc	0.03179
279_R_Default -- 155_L_DorsalAttn	0.00099	116_L_Default -- 168_R_FrontoParietal	0.00479	162_R_Default -- 22_L_CinguloOperc	0.03835
127_L_Default -- 249_R_CinguloOperc	0.00178	157_L_Default -- 21_L_CinguloOperc	0.00506	1_L_Default -- 22_L_CinguloOperc	0.04007
127_L_Default -- 84_L_CinguloOperc	0.00718	322_R_Default -- 236_R_DorsalAttn	0.00773	1_L_Default -- 245_R_CinguloOperc	0.03492
116_L_Default -- 249_R_CinguloOperc	0.00798	322_R_Default -- 84_L_CinguloOperc	0.02272	157_L_Default -- 185_R_CinguloOperc	0.04380
279_R_Default -- 219_R_CinguloOperc	0.00727	127_L_Default -- 110_L_DorsalAttn	0.03360	315_R_Default -- 63_L_CinguloOperc	0.04762
279_R_Default -- 318_R_CinguloOperc	0.00884	116_L_Default -- 236_R_DorsalAttn	0.03389	1_L_Default -- 274_R_CinguloOperc	0.04667
322_R_Default -- 84_L_CinguloOperc	0.00814	145_L_Default -- 249_R_CinguloOperc	0.03924	290_R_Default -- 318_R_CinguloOperc	0.04538
116_L_Default -- 219_R_CinguloOperc	0.01580	157_L_Default -- 180_R_CinguloOperc	0.04309	162_R_Default -- 63_L_CinguloOperc	0.04373
279_R_Default -- 275_R_DorsalAttn	0.03037	290_R_Default -- 63_L_CinguloOperc	0.04601	220_R_Default -- 76_L_CinguloOperc	0.04642
117_L_Default -- 275_R_DorsalAttn	0.02885	156_L_Default -- 21_L_CinguloOperc	0.04628	94_L_Default -- 181_R_CinguloOperc	0.04692
290_R_Default -- 155_L_DorsalAttn	0.03007	127_L_Default -- 236_R_DorsalAttn	0.04673	315_R_Default -- 223_R_CinguloOperc	0.04444
278_R_Default -- 275_R_DorsalAttn	0.03252			127_L_Default -- 249_R_CinguloOperc	0.05057
279_R_Default -- 84_L_CinguloOperc	0.03377			127_L_Default -- 84_L_CinguloOperc	0.04764
145_L_Default -- 249_R_CinguloOperc	0.03342			1_L_Default -- 318_R_CinguloOperc	0.04708
116_L_Default -- 318_R_CinguloOperc	0.03636			162_R_Default -- 76_L_CinguloOperc	0.04654
152_L_Default -- 275_R_DorsalAttn	0.03710			322_R_Default -- 84_L_CinguloOperc	0.04524
322_R_Default -- 236_R_DorsalAttn	0.04515				
150_L_Default -- 236_R_DorsalAttn	0.04503				
116_L_Default -- 168_R_FrontoParietal	0.04698				
322_R_Default -- 106_L_DorsalAttn	0.04916				

Supplementary Table 4. Group, age, and sex effects of average negative connectivity between task positive and task negative (default) brain systems. ROIs and network labels are defined by the Gordon parcellation (Gordon et al., 2014). All p-values represent the FDR corrected significance for each main effect in a linear mixed effects model including all three parameters (model 1).

Supplementary References

Conners, C. K. (2008). *Conners 3rd Edition Manual*. (M. Rayes & V. Hartawidjojo, Eds.). Toronto: Multi-Health Systems Inc.

Cornblatt, B. A., Risch, N. J., Faris, G., Friedman, D., & Erlenmeyer-Kimling, L. (1988). The Continuous Performance Test, identical pairs version (CPT-IP): I. New findings about sustained attention in normal families. *Psychiatry Research*, *26*(2), 223–38. Retrieved from http://www.ncbi.nlm.nih.gov/pubmed/3237915

Curko Kera, E. A., Marks, D. J., Berwid, O. G., Snatra, A., & Halperin, J. M. (2004). Self-report and objective measures of ADHD-related behaviors in parents of preschool children at risk for ADHD. *CNS Spectr*, *9*(9), 639–647.

Desikan, R. S., Segonne, F., Fischl, B., Quinn, B. T., Dickerson, B. C., Blacker, D., … Killiany, R. J. (2006). An automated labeling system for subdividing the human cerebral cortex on MRI scans into gyral based regions of interest. *Neuroimage*, *31*(3), 968–980. https://doi.org/S1053-8119(06)00043-7 [pii]10.1016/j.neuroimage.2006.01.021

DuPaul, G., Power, T., Anastopoulos, A., & Reid, R. (1998). *ADHD Rating Scales-IV: Checklists, Norms and Clinical Interpretation*. New York: Guilford Press.

Fair, D. A., Nigg, J. T., Iyer, S., Bathula, D., Mills, K. L., Dosenbach, N. U. F., … Milham, M. P. (2012). Distinct neural signatures detected for ADHD subtypes after controlling for micro-movements in resting state functional connectivity MRI data. *Frontiers in Systems Neuroscience*, *6*(February), 80. https://doi.org/10.3389/fnsys.2012.00080

Fischl, B., & Dale, A. M. (2000). Measuring the thickness of the human cerebral cortex from magnetic resonance images. *Proc Natl Acad Sci U S A*, *97*(20), 11050–11055. https://doi.org/10.1073/pnas.200033797

Fischl, B., Sereno, M. I., & Dale, A. M. (1999). Cortical surface-based analysis. II: Inflation, flattening, and a surface-based coordinate system. *Neuroimage*, *9*(2), 195–207. Retrieved from http://www.ncbi.nlm.nih.gov/entrez/query.fcgi?cmd=Retrieve&db=PubMed&dopt=Citation&list_uids=9931269

Fox, M. D., Zhang, D., Snyder, A. Z., & Raichle, M. E. (2009). The global signal and observed anticorrelated resting state brain networks. *Journal of Neurophysiology*, *101*(6), 3270–3283. https://doi.org/90777.2008 [pii]10.1152/jn.90777.2008

Friston, K. J., Williams, S., Howard, R., Frackowiak, R. S., & Turner, R. (1996). Movement-related effects in fMRI time-series. *Magn Reson Med*, *35*(3), 346–355. Retrieved from http://www.ncbi.nlm.nih.gov/pubmed/8699946

Glasser, M. F., Sotiropoulos, S. N., Wilson, J. A., Coalson, T. S., Fischl, B., Andersson, J. L., … Jenkinson, M. (2013). The minimal preprocessing pipelines for the Human Connectome Project. *NeuroImage*, *80*, 105–24. https://doi.org/10.1016/j.neuroimage.2013.04.127

Goodman, R. (1997). The Strengths and Difficulties Questionnaire: a research note. *J Child Psychol Psychiatry*, *38*(5), 581–586. Retrieved from http://www.ncbi.nlm.nih.gov/pubmed/9255702

Gordon, E. M., Laumann, T. O., Adeyemo, B., Huckins, J. F., Kelley, W. M., & Petersen, S. E. (2014). Generation and Evaluation of a Cortical Area Parcellation from Resting-State Correlations. *Cerebral Cortex (New York, N.Y. : 1991)*, *26*(1), 288–303. https://doi.org/10.1093/cercor/bhu239

Gordon, E. M., Laumann, T. O., Adeyemo, B., Huckins, J. F., Kelley, W. M., & Petersen, S. E. (2016). Generation and Evaluation of a Cortical Area Parcellation from Resting-State Correlations. *Cerebral Cortex*, *26*(1), 288–303. https://doi.org/10.1093/cercor/bhu239

Gotts, S. J., Saad, Z. S., Jo, H. J., Wallace, G. L., Cox, R. W., & Martin, A. (2013). The perils of global signal regression for group comparisons: A case study of Autism Spectrum Disorders. *Front Hum Neurosci*, *7*. https://doi.org/10.3389/fnhum.2013.00356

Grayson, D. S., Bliss-Moreau, E., Machado, C. J., Bennett, J., Shen, K., Grant, K. A., … Amaral, D. G. (2016). The Rhesus Monkey Connectome Predicts Disrupted Functional Networks Resulting from Pharmacogenetic Inactivation of the Amygdala. *Neuron*, *91*(2), 453–66. https://doi.org/10.1016/j.neuron.2016.06.005

Greve, D. N., & Fischl, B. (2009). Accurate and robust brain image alignment using boundary-based registration. *NeuroImage*, *48*(1), 63–72.

https://doi.org/10.1016/j.neuroimage.2009.06.060
Halperin, J. M., Sharma, V., Greenblatt, E., & Schwartz, S. T. (1991). Assessment of the continuous performance test: Reliability and validity in a nonreferred sample. *Psychological Assessment: Journal of Consulting and Clinical Psychology*, *3*, 603–608.
Huang-Pollock, C. L., Karalunas, S. L., Tam, H., & Moore, A. N. (2012). Evaluating vigilance deficits in ADHD: a meta-analysis of CPT performance. *Journal of Abnormal Psychology*, *121*(2), 360–71. https://doi.org/10.1037/a0027205
Jenkinson, M., Beckmann, C. F., Behrens, T. E. J., Woolrich, M. W., & Smith, S. M. (2012). FSL. *NeuroImage*, *62*(2), 782–790. https://doi.org/10.1016/j.neuroimage.2011.09.015
Keller, C., Bickel, S., Honey, C., Groppe, D., Entz, L., Craddock, R. C., … Mehta, A. D. (2013). Neurophysiological Investigation of Spontaneous Correlated and Anticorrelated Fluctuations of the BOLD Signal. *Journal of Neuroscience*, *33*(15), 6333–6342. https://doi.org/10.1523/JNEUROSCI.4837-12.2013
Miranda-Domínguez, Ó., Mills, B. D., Grayson, D., Woodall, A., Grant, K. A., Kroenke, C. D., & Fair, D. A. (2014). Bridging the gap between the human and macaque connectome: a quantitative comparison of global interspecies structure-function relationships and network topology. *The Journal of Neuroscience : The Official Journal of the Society for Neuroscience*, *34*(16), 5552–5563. https://doi.org/10.1523/JNEUROSCI.4229-13.2014
Murphy, K., Birn, R. M., Handwerker, D. a, Jones, T. B., & Bandettini, P. a. (2009). The impact of global signal regression on resting state correlations: are anti-correlated networks introduced? *NeuroImage*, *44*(3), 893–905. https://doi.org/10.1016/j.neuroimage.2008.09.036
Murphy, K., & Fox, M. D. (2016). Towards a Consensus Regarding Global Signal Regression for Resting State Functional Connectivity MRI. *Neuroimage*. https://doi.org/10.1016/j.neuroimage.2016.11.052
Orvaschel, H., Lewinsohn, P. M., & Seeley, J. R. (1995). Continuity of psychopathology in a community sample of adolescents. *J Am Acad Child Adolesc Psychiatry*, *34*(11), 1525–1535. Retrieved from http://www.ncbi.nlm.nih.gov/entrez/query.fcgi?cmd=Retrieve&db=PubMed&dopt=Citation&list_uids=8543521
Parasuraman, R., & Davies, D. R. (1977). A Taxonomic Analysis of Vigilance Performance. In *Vigilance* (pp. 559–574). Boston, MA: Springer US. https://doi.org/10.1007/978-1-4684-2529-1_26
Power, J., Barnes, K., Snyder, A., Schlaggar, B., & Petersen, S. (2012). Spurious but systematic correlations in functional connectivity MRI networks arise from subject motion. *Neuroimage*, *59*(3), 2142–2154. https://doi.org/10.1016/j.neuroimage.2011.10.018
Power, J., Mitra, A., Laumann, T., Snyder, A., Schlaggar, B., & Petersen, S. (2013). Methods to detect, characterize, and remove motion artifact in resting state fMRI. *Neuroimage*. https://doi.org/10.1016/j.neuroimage.2013.08.048
Power, J., Mitra, A., Laumann, T., Snyder, A., Schlaggar, B., & Petersen, S. (2014). Methods to detect, characterize, and remove motion artifact in resting state fMRI. *Neuroimage*, *84*, 320–341. https://doi.org/10.1016/j.neuroimage.2013.08.048
Satterthwaite, T. D., Elliott, M. A., Gerraty, R. T., Ruparel, K., Loughead, J., Calkins, M. E., … Wolf, D. H. (2013). An improved framework for confound regression and filtering for control of motion artifact in the preprocessing of resting-state functional connectivity data. *Neuroimage*, *64*, 240–256. https://doi.org/10.1016/j.neuroimage.2012.08.052

Satterthwaite, T., Wolf, D., Loughead, J., Ruparel, K., Elliott, M., Hakonarson, H., … Gur, R. (2012). Impact of in-scanner head motion on multiple measures of functional connectivity: relevance for studies of neurodevelopment in youth. *Neuroimage*, *60*(1), 623–632. https://doi.org/10.1016/j.neuroimage.2011.12.063

Schölvinck, M. L., Maier, A., Ye, F. Q., Duyn, J. H., & Leopold, D. A. (2010). Neural basis of global resting-state fMRI activity. *Proceedings of the National Academy of Sciences of the United States of America*, *107*(22), 10238–10243. Retrieved from http://www.ncbi.nlm.nih.gov.beckerproxy.wustl.edu/pubmed/20439733

Sergeant, J. A., Oosterlaan, J., & van der Meere, J. J. (1999). *Information processing and energetic factors in attention deficit/hyperactivity disorder*. (H. C. Quay & A. E. Hogan, Eds.), *Handbook of disruptive behavior disorders*. New York: Kluwer/Plenum Publishers.

Siegel, J. S., Mitra, A., Laumann, T. O., Seitzman, B. A., Raichle, M., Corbetta, M., & Snyder, A. Z. (2016). Data Quality Influences Observed Links Between Functional Connectivity and Behavior. *Cerebral Cortex (New York, N.Y. : 1991)*. https://doi.org/10.1093/cercor/bhw253

Smith, S. M., Jenkinson, M., Woolrich, M. W., Beckmann, C. F., Behrens, T. E. J., Johansen-Berg, H., … Matthews, P. M. (2004). Advances in functional and structural MR image analysis and implementation as FSL. *NeuroImage*, *23 Suppl 1*, S208--19. https://doi.org/10.1016/j.neuroimage.2004.07.051

Stanislaw, H., & Todorov, N. (1999). Calculation of signal detection theory measures. *Behavior Research Methods, Instruments, & Computers*, *31*(1), 137–149. https://doi.org/10.3758/BF03207704

Tucha, L., Tucha, O., Walitza, S., Sontag, T. A., Laufkötter, R., Linder, M., & Lange, K. W. (2009). Vigilance and sustained attention in children and adults with ADHD. *Journal of Attention Disorders*, *12*(5), 410–21. https://doi.org/10.1177/1087054708315065

Wechsler, D. (2003). *Wechsler Intelligence Scale for Children* (Fourth). San Antonio, TX: The Psychological Corporation.

Willcutt, E. G., Doyle, A. E., Nigg, J. T., Faraone, S. V, & Pennington, B. F. (2005). Validity of the executive function theory of attention-deficit/hyperactivity disorder: a meta-analytic review. *Biological Psychiatry*, *57*(11), 1336–1346. https://doi.org/10.1016/j.biopsych.2005.02.006

WU-Minn, H. (2014). 500 Subjects+ MEG2 Data Release: Reference Manual.
